# Vaginal small-bowel evisceration 15 years post wertheim-hysterectomy: a case report and review of literature

**DOI:** 10.3389/fsurg.2026.1782864

**Published:** 2026-04-21

**Authors:** Florian Högler, Filipp Sokolovski, Sandra Raab, Lena Rossetti, Peter Oppelt, Andreas Shamiyeh

**Affiliations:** 1 Medical Faculty, Johannes Kepler University (JKU), Linz, Austria; 2 Clinic for General and Visceral Surgery, Kepler University Clinic, Linz, Austria; 3 Clinic for Gynaecology, Obstetrics and Gynaecological Endocrinology, Kepler University Clinic, Linz, Austria

**Keywords:** case report, complication, hysterectomy, laparoscopy, rare, small-bowel evisceration, vaginal cuff

## Abstract

Vaginal cuff dehiscence with evisceration (VCDE) is a rare but severe complication of hysterectomy with potentially lethal consequences requiring rapid surgical intervention. Due to the high frequency of hysterectomies, healthcare professionals should be aware of the condition and its aetiology, clinical presentation and precipitating factors to identify patients at risk. We report the case of a 74-year-old woman undergoing bowel resection due to VCDE 15 years post Wertheim-procedure.

## 1 Introduction

Vaginal cuff dehiscence (VCD) is a rare but potentially fatal complication following total hysterectomy. It is characterised by the postoperative opening of the vaginal stump resulting in a direct connection from the peritoneal cavity to the vagina. Clinical presentation spans a wide range from vaginal discharge and pelvic pain to extensive gastrointestinal bleeding, severe abdominal/pelvic pain, and evisceration of abdominal contents, referred to as a vaginal cuff dehiscence with evisceration (VCDE). Visible evisceration is seen in only a third of patients (1). Whilst the distal ileum is the most frequently eviscerated organ, the prolapse of other abdominal organs such as the sigmoid colon, omentum, appendix and adnexa uteri has been reported (2). In all cases, it is crucial to assess the vitality of the prolapsed tissue (3).

The aim of this paper is to report the case of a patient presenting with small-bowel evisceration secondary to VCD, who experienced a complicated postoperative course. Additionally, a review of the existing literature of relevant PubMed articles was performed to identify the prevalence of VCDE by each type of surgery, key precipitating factors, main symptoms and complications of VCDE-repair, comparing our approach with existing literature. We hope to add valuable data in order to determine optimal treatment options in this rare condition. This case has been reported in line with the SCARE criteria (4).

## 2 Case presentation

**Figure 1:**
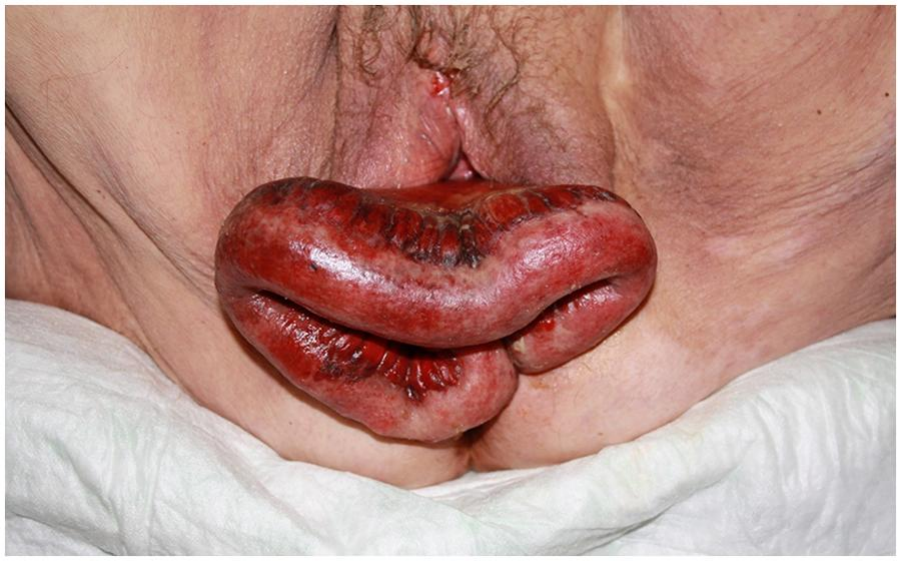
30 cm of small bowel and mesentery evisceration through the vaginal cuff dehiscence with ischaemic presentation.

A 74-year-old Caucasian woman presented to the emergency department after being found by her home carer in a neglected and unattended state with strong hypogastric pain, emesis, enuresis and visible vaginal bowel protrusion with haemorrhagic discharge. She reported that the vaginal protrusion occurred during strained defecation. Lab values showed increased leucocyte count at 14.9 G/L (3.9–8.8), elevated C-reactive protein (CRP) at 118 mg/L (<5.0) and acute kidney failure with increased creatinine level of 2.53 mg/dL (0.67–1.17) and blood urea nitrogen (BUN) levels of 52 mg/dL (8–23). Evaluation by the on-call gynaecologist and general surgeon revealed a 50 cm evisceration of suspectedly incarcerated small-bowel (Figure 1), which was immediately covered with saline-soaked gauze. A digital rectal exam showed no involvement of the rectovaginal septum.

Fifteen years prior she underwent a Wertheim-Meigs-hysterectomy, bilateral salpingo-oophorectomy (BSO) with pelvic lymph node dissection, appendectomy and panniculectomy, due to endometrial cancer with infiltration of the cervix (pT1a pN0 R0) (5). The vaginal cuff was closed via a continuous running stitch. She had a medical history significant of congestive heart failure, atrial fibrillation, COPD and type 2 Diabetes, alongside a BMI of 36.7 kg/m^2^.

An emergency exploratory laparotomy was performed with vaginal reduction of the incarcerated small bowel. The vaginal cuff was completely dehiscent and no apparent reason for VCD could be determined, with no macroscopic signs indicative of neoplasia, which was also histologically confirmed by vaginal cuff biopsy. The cuff defect was closed using Vicryl 1/0 interrupted sutures by the consultant gynaecologist. Inspection of the incarcerated intestinal loops revealed 50 cm of ileal necrosis just proximal of the ileocecal valve. Therefore, an ileocecal resection (mechanical side-to-side ileoascendostomy) and lavage/drainage was performed. After insertion of a Robinson-Drain into the rectouterine pouch, the peritoneum and abdominal wall were closed. Perioperatively, the patient received intravenous antibiosis with piperacillin/tazobactam, short-acting insulin for hyperglycaemia-control and intravenous furosemide for pedal oedema. Histological examination of the resected ileocecal segment showed mucosal necrosis and signs of fibrinous-purulent peritonitis. Oral and aboral margins were vital. No signs of malignancy were present in the bowel specimen or vaginal biopsies.

The postoperative course proved complicated. On the 10th postoperative day anastomotic leakage occurred, requiring resection and re-creation of the ileoascendostomy (manual side-to-side). Due to extensive damage and contamination of the abdominal wall, some fascia and subcutaneous tissue also had to be resected and abdominal dressing therapy was initiated. Fascia closure and subcutaneous vacuum-assisted closure (VAC) were performed after 5 days, and after multiple changes of the VAC system, complete wound closure was obtained after 18 days. After 65 days the patient was transferred to in-patient rehabilitation service, which she was discharged from after another 21 days, resulting in a total in-hospital stay of 86 days. Upon discharge, the patient was lost to follow-up.

## 3 Discussion and literature review

A systematic PubMed search was conducted in October 2024 to identify reported cases of VCDE, using the search terms “vaginal cuff dehiscence with evisceration” (60 results), “vaginal evisceration post hysterectomy” (24 results), “vaginal evisceration after hysterectomy” (104 results) and “vaginal cuff dehiscence” (221 results). The following filters were applied: “English” and “humans”, to exclude animal-conducted studies. Only articles with abstracts and full texts containing sufficient information, accessible through the university's credentials (Johannes Kepler University, Linz, Austria) were screened and used in data analysis.

Using these criteria, 66 articles were identified and included in the analysis, comprising a total of 87 reported patients (see Additional File 1). This cohort represents all published cases of VCDE available up to October 2024 that contained sufficient clinical information for our analysis. The literature findings are summarized in Table 1.

**Table 1:** Results of data analysis for patients with VCDE.

Mean age (^a^ = 87)		52.6 years
Median time between hysterectomy and VCDE (^a^ = 87)		4.7 months
Symptoms (^a^ = 82)	Abdominal pain	63.8%
	Vaginal protrusion	50%
	Vaginal discharge	37.8%
	Asymptomatic	2.4%
Precipitating event (^a^ = 75)	Coitus	54.7%
	Abdominal strain	29.3%
	Spontaneous	9.3%
	Not reported	5.6%
VCDE frequency by surgical approach of hysterectomy (^a^ = 86)	Open abdominal	38.4%
	Laparoscopic	52.3%
	Vaginal approach	9.3%
Total complication rate post VCDE surgery (^a^ = 75)		9.3%

a indicates the number of patients with sufficient information to be included for calculation.

Although hysterectomy is a common procedure, VCD is a very rare complication. Its prevalence has been reported within the range of 0.1%–5.4% in a systematic review by Moens, Buonomo and De Sutter (6) with intestinal evisceration occurring in 35%–67% of these cases (7). VCDE is predominantly seen in postmenopausal women, representing approximately 70% of cases, a phenomenon associated with decreased vaginal wall vascularity and atrophy (8). Consistent with this, the patient described in this case report was also postmenopausal.

Knowledge of the menopausal status is important, as precipitating events differ in pre- and postmenopausal women. Whilst sexual intercourse has been observed to be the most common inducing incident in premenopausal women, intraabdominal increases of pressure, commonly coughing, strained defecation and sneezing, were identified as leading causes in postmenopausal women (9). In line with this, the precipitating event leading to VCDE in our patient, an elderly postmenopausal woman, was strained defecation. Our literature review showed intercourse to be the most common precipitating event overall (54.6%), mainly due to many patients not adhering to the 6–8-week period of postoperative abstinence. This was followed by raised intraabdominal pressure (29.3%) and spontaneous occurrence (9.3%). We believe that it is important to inform patients about the potential risk of VCD with or without evisceration, and educate them about adequate fluid intake, fibre-rich diet and laxantia, as well as the importance of adhering to post-operative abstinence.

We calculated the median time for VCDE occurrence to be 4.7 months post-hysterectomy, ranging from three days to 40 years. In our patient VCDE occurred 15 years after abdominal hysterectomy. Patient risk factors are mode of hysterectomy, increased age, pelvic floor weakness with chronic prolapse, chronic kidney disease and factors associated with wound healing such as chronic steroid use, poorly controlled diabetes, hypoestrogenism and malnutrition (10). However, risk factors have generally proven difficult to identify, predominantly due to the limited body of available evidence. This data scarcity is not only due to the low prevalence of VCDE, but also a result of little variation in publication type, as the majority are case reports (11).

The mode of hysterectomy has also been observed to influence the incidence of VCD, with the highest rates reported following total laparoscopic hysterectomy (TLH, 5.4%) and the lowest following vaginal hysterectomy (VH, 0.1%) (6). Despite the scant data, the same trend was observed by Tsakona et al. (7), showing abdominal and vaginal hysterectomies to be generally safer in terms of the occurrence of VCD. Our analysis also supported this trend.

These findings suggest that laparoscopic surgical techniques could thus be revised or modified to reduce the incidence of VCD. Siedhoff et al. demonstrated this by evaluating the use of bidirectional barbed sutures instead of braided sutures, showing a reduction from 4.2% to 0% (*n* = 387, *p* = 0.008), while also decreasing postoperative bleeding, granulation tissue formation and cellulitis (12). Other studies also support this trend: Fenske et al. (13) report a 74% risk reduction of VCD by using unidirectional barbed sutures rather than interrupted Polyglactin sutures, and Rettenmaier et al. observed no dehiscence in patients with a barbed suture cuff repair (14). Notably, the follow-up periods in these studies vary greatly and, in some cases, remain unspecified. In our cohort, insufficient documentation of suture material and technique prevented a similar analysis, highlighting the importance of detailed reporting to accurately assess postoperative outcomes.

In terms of clinical management, it is crucial to immediately evaluate the vitality of eviscerated intestinal tissue, which is most commonly the distal ileum. Immediate reduction or covering with sterile gauze is required to reduce the risk of necrosis, alongside administration of broad-spectrum antibiotics and intravenous fluids until transfer to the operating theatre (2). To rapidly recognize VCDE, knowledge of possible key primary symptoms is vital. In our literature search, we found only 50% of patients with evisceration to present with a visible protrusion, whilst the majority (63.4%) reported abdominal pain and/or vaginal discomfort, as well as 37.8% experiencing atypical vaginal discharge or bleeding.

Historically, an exploratory laparotomy was the only option for VCDE repair, however it is worth noting that successful transvaginal approaches have been reported and are now gaining ground (15). A vaginal approach is primarily used when no signs of peritonitis, ischemic injury or strangulation are present, but as of date, no unanimous decision on the optimal approach for VCDE repair exists (16). There are also reports of laparoscopic approaches for VCDE repair in the literature (6). In our patient, there was a clear indication for exploratory laparotomy, due to the visibly ischemic small-bowel loops and mesentery upon gynaecological and surgical examination.

## 4 Conclusion

Vaginal cuff dehiscence with evisceration (VCDE) is a rare and potentially lethal surgical emergency secondary to total hysterectomy, which requires rapid management to prevent further complications such as bowel necrosis and sepsis. Whilst risk factors are difficult to identify, our case report shows that this condition should be routinely suspected in women with a history of hysterectomy, regardless of how recently the procedure was performed.

Our literature review highlights the importance of postoperative patient management. We believe the rate of VCDE could be reduced by improving patient education in postoperative behaviour (especially adherence to the recommended six-week abstinence period), and by minimising defecatory strain through adequate fluid intake, laxatives and high-fibre-products. Our review also found higher VCD rates after laparoscopic hysterectomy, consistent with existing literature, suggesting the necessity to modify laparoscopic procedures, such as using bidirectional sutures, to reduce incidence.

Due to the low prevalence of VCDE and paucity of published data, establishing a consensus on the management of this condition remains a challenge. We hope that this paper may act as a chaperone in clinical management of patients with VCDE.

## Data Availability

The original contributions presented in the study are included in the article/Supplementary Material, further inquiries can be directed to the corresponding author.
